# The Use of Computational Approaches in the Discovery and Mechanism Study of Opioid Analgesics

**DOI:** 10.3389/fchem.2020.00335

**Published:** 2020-05-15

**Authors:** Bangyi Zhao, Wei Li, Lijie Sun, Wei Fu

**Affiliations:** ^1^Department of Medicinal Chemistry, School of Pharmacy, Fudan University, Shanghai, China; ^2^Shijiazhuang No. 4 Pharmaceutical Co., Ltd., Shijiazhuang Economic and Technological Development Zone, Shijiazhuang, China

**Keywords:** opioid receptor, analgesics, computer-aided drug design, molecular dynamics, agonist mechanism, G-protein-biased activation

## Abstract

Opioid receptors that belong to class A G protein-coupled receptors (GPCRs) are vital in pain control. In the past few years, published high-resolution crystal structures of opioid receptor laid a solid basis for both experimental and computational studies. Computer-aided drug design (CADD) has been established as a powerful tool for discovering novel lead compounds and for understanding activation mechanism of target receptors. Herein, we reviewed the computational-guided studies on opioid receptors for the discovery of new analgesics, the structural basis of receptor subtype selectivity, agonist interaction mechanism, and biased signaling mechanism.

## Introduction

G protein-coupled receptors (GPCRs), also known as hepta-helical receptors, are characterized as α-helix domains which span the cell membrane seven times (King et al., 2003). As a large family of membrane proteins, GPCRs are targeted by about 40% of all modern drugs (Overington et al., 2006). Opioid receptors are a subfamily of GPCRs and are responsible for powerful analgesic effects. They are mainly divided into four subtypes, μ, δ, κ, and nociceptin/orphanin FQ (N/OFQ or NOP). Lately, Claff et al. (2019) reported the first active crystal structure of the δ opioid receptor. This will enhance the future structure-based development of opioid agonists along with some previously revealed crystal structures. Inspired by the progress in the determination of opioid receptor, here we summarize the recent development in the use of computational approaches in the discovery and mechanism study of opioid analgesics.

When an agonist binds to the opioid receptor, it will activate G proteins, inhibit adenylate cyclase, and promote the extrusion of potassium ions. These events can finally lead to the hyperpolarization of cell membranes (Snyder, 1977; Simon and Hiller, 1978). These events also block or minimize the transmission of painful stimuli and raise the pain threshold. For centuries, opioid analgesics have been used to manage acute and chronic pain with the level from moderate to severe. These analgesics, like morphine, fentanyl, etc., mainly target the μ opioid receptor. These drugs have strong analgesic effects that cannot be replaced by other analgesic drugs, and therefore, become the first-line treatment of cancer pain, surgical pain, and other kinds of pain. However, usage of these drugs has brought about obvious side effects such as constipation, drug tolerance, addiction, and respiratory depression. These unwanted side effects put limits on their clinical use. Also reported are some other analgesics in clinical use with relatively less addiction. These include tramadol, buprenorphine, butorphanol, and dezocine. The analgesics with less addiction are mostly targeted to multiple opioid receptors. For example, butorphanol is a partial agonist of μ and κ receptors, while dezocine is recently recognized as an agonist of μ opioid receptor and an inhibitor of norepinephrine transporter (μOR-NRI) (Wang et al., 2017).

However, there is strong clinical need for new analgesics with less side effects. As inspired, researchers are generating novel ideas, of which are continuously being evaluated. These include the design of κ or δ opioid selective agonists, new bifunctional μ receptor agonists (μ/δ, μOR-NRI, NOP/μOR) and G protein-biased ligand. The κ opioid agonists can offer effective analgesia without causing typical opioid-related adverse effects, which means selective κ opioid can serve agonists as analgesics (Assana et al., 2014; Grechko et al., 2016; Zaitseva et al., 2018). Similarly, the δ opioid receptor (δOR) has also been identified as a potent target for the discovery of novel analgesic drugs, with less side effects and better analgesia in animal models (Desmeules et al., 1993; Fraser et al., 2000; Brandt et al., 2001; Mika et al., 2001). After studying the interaction between μ receptor and other targets, researchers raised the concept of bifunctional μ receptor agonists. They found that the δ receptor agonists can enhance the analgesic effect of the μ receptor agonists, while the δ receptor antagonists can significantly improve or even completely block the side effects of addiction, tolerance, and respiratory inhibition caused by the μ receptor agonists (Abdelhamid et al., 1991; Porreca et al., 1992). They also found that the activation of NOP can reduce the reinforcing effects by the way of decreasing dopamine levels (Liu et al., 2001). Additional study of opioid receptor function has demonstrated that G protein conjugation pathway of μ opioid receptor mainly induces analgesic effect, while the β-arrestin pathway can lead to side effects such as respiratory depression, nausea, and constipation (Violin et al., 2014). These new concepts need to be tested with extensive follow-up studies, and computational study is an appropriate and efficient test.

The concept of computer-aided drug design (CADD) firstly appeared in 1981 (Van Drie, 2007) and immediately was used extensively in the drug discovery. Since then, CADD has intensively improved the efficiency of traditional high-throughput screening (HTS) method, and it has significantly lowered the cost while retaining the same level of lead compound discovery (Sliwoski et al., 2014). Nowadays, CADD methods have been widely used in the modern drug discovery and mechanism studies on all drug-targeted proteins. In this review, we will focus on the applications of computational approaches in these topics: (1) discovering opioid analgesics; (2) the molecular mechanisms of opioid subtype selectivity; (3) the mechanism of the opioid receptor activation, and specifically, the G protein-biased activation.

## Computational Approaches in The Discovery of Opioid Analgesics

### Structure-Based Virtual Screening

Structure-based virtual screening (SBVS) starts from the three-dimensional (3D) structure of a target receptor to design potential active compounds (Cavasotto and Orry, 2007) through the molecular docking operations and reasonable scoring for a library of compounds. Due to various experimental difficulties in determining the structures of GPCR, the number of determined GPCR structures is still small, but keeps growing. As such, homology modeling offers a relatively reasonable strategy to build the model structure of the target receptor based on its amino acids sequence and an available homologous structure (as a template) of a related protein.

As a main tool, molecular docking commonly applied used in SBVS to evaluate the interactions between ligands and a particular receptor in order to rank the binding affinities of these ligands. The ligands are docked into the active site of a receptor through conformational search and a pre-built scoring function during the virtual screening. Researchers have developed many conformational search algorithms, including molecular dynamics (MD) simulation, systematical methods, Monte Carlo (MC) search, and genetic algorithms (GAs). Scoring functions include the molecular mechanics-based scoring functions, the empirical scoring functions, the knowledge-based scoring functions, and the consensus scoring functions (Sliwoski et al., 2014). For a particular receptor protein, both a feasible search algorithm and an accurate scoring function have to be carefully tested and selected.

**Figure 1:**
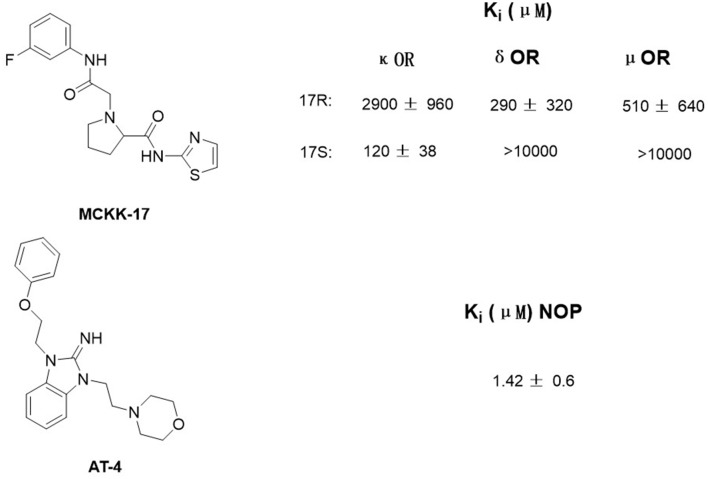
Structure of MCKK-17 and AT-4 found by structure-based virtual screening (SBVS) and their Ki to respective receptors.

Luckily, many efforts have been made in determining the crystal structures of opioid receptors. In 2012, studies revealed the inactive crystal structures of all four opioid receptor subtypes, i.e., the μ receptor (Manglik et al., 2012), the δ receptor (Granier et al., 2012), the κ receptor (Wu et al., 2012), and N/OFQ (Thompson et al., 2012) receptor. Later in 2015, the crystal structure of active μ (O'Connor et al., 2015) and κ (Huang et al., 2015) opioid receptors were also resolved. In 2019, δOR's active crystal structure has been finally obtained (Claff et al., 2019). These crystal structures can be directly used for SBVS. Aiming at discovering potent non-addictive analgesics, Negri et al. (2013) virtually screened through docking 4,554,059 compounds of the ZINC database to inactive crystal structure of κ opioid receptor and found a selective novel agonist, MCKK-17S (Figure 1, top). In 2014, Daga et al. (2014) firstly applied SBVS to find novel hits for the nociception opioid receptor. In that study, they built an active state homology model of NOP based on the antagonist-bound crystal structure and refined it by the enrichment analysis. Shape-based approaches were also applied to improve the hit rate of the screening, and a new chemical scaffold was finally discovered. Among these newly discovered compounds, AT4 showed the best receptor affinity (Ki = 1.42 ± 0.6 μM) (Figure 1, bottom).

Although a number of crystal structures of opioid receptor have been reported, it is still a challenge to figure out the actual active conformation of the receptor when docking it with different ligands. It has been demonstrated that the affinities predicted for the μ opioid receptor by docking failed to significantly correlate with the reported experimental values (Cui et al., 2013). This indicates that the receptor may go through conformational transitions upon ligand binding. Thus, the results of SBVS based on docking may be some kind of misleading.

### Ligand-Based Virtual Screening

Ligand-based virtual screening (LBVS) method compares known active compounds of the target receptor with compounds in a database like SPECS and ZINC and select ligands with better activity. Compared to SBVS, the LBVS method is usually faster and does not depend on the information about the structure of the target receptor (Lemmen and Lengauer, 2000). Based on the complexity of ligand structure information that is used, LBVS can be divided into three classes, one-dimensional (1D), two-dimensional (2D), and 3D. Among them, 3D methods have better performance since it takes the conformational flexibility of compounds into consideration. However, 3D methods are challenging as the bioactive conformation of a ligand frequently does not match the lowest-energy conformation in many LBVS cases (Leach and Gillet, 2007). They can be further divided into five classes as atomic distance-based, surface-based, Gaussian function-based, field-based, and pharmacophore-based methods (Shin et al., 2015).

As early as in the year 2008, Zhang et al. (2009) have generated two pharmacophore models for the κ-agonists using Catalyst/HypoGen and Phase respectively. These models could predict the structure-activity relationship and help to develop some new compounds. In the paper by Zhang et al., they also evaluated and compared the two models. These two models shared one hydrogen-bond receptor and one positive ionizable function and differ only in their definition of the hydrophobic point and aromatic ring features.

So far, the obvious weakness of LBVS method is its ability to identify the bioactive conformation of a ligand. As it also depends on the structure of the discovered active compounds, LBVS method cannot bring about very novel and diversified active structures.

## Computational Approaches in Mechanism Study of Opioid Receptors

### Molecular Docking to Illustrate Opioid Receptor Selectivity

**Figure 2:**
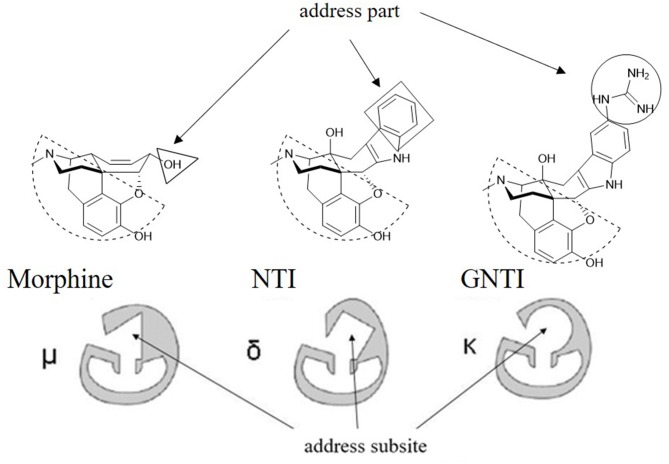
Proposed “message–address” concept in selective opioid receptor ligand morphine, naltrindole (NTI), and 5′-guanidinonaltrindole (GNTI).

As reported, the κ and δ opioid receptors have been recognized as potent targets to develop analgesic drugs with less side effects. It has been found that some drugs in the clinic showed less side effects and are proved to interact with more than one subtype of opioid receptors. Compounds as butorphanol and buprenorphine are typical examples. To better understand the molecular mechanism of such multi-target ligands and subtype selectivity, researchers proposed the classic “message–address” concept to explore the binding mode between the opioid receptors and their ligands (Schwyzer, 1977). That is, the “message” part of the ligand is responsible for receptor recognition and affinity, while the “address” part of the ligand determines the selectivity and/or potency (Figure 2). It was successfully applied in the discovery of δOR antagonist naltrindole (Portoghese et al., 1990). The “message” and “address” parts interact with different residues at the active site of opioid receptor. Different subtypes have similar “message” subsites but totally different “address” subsites. This concept was widely used not only to explain the selectivity of active compounds but also to design better selective molecules through computer-aided molecular docking and/or MD simulations.

#### The κ Opioid Receptor

**Figure 3:**
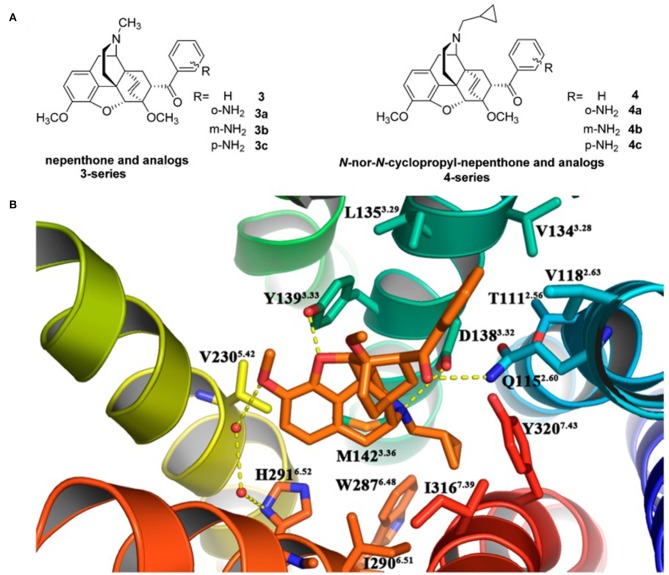
**(A)** Design for novel κOR agonists from nepenthone (3-series) and N-nor-N-cyclopropylmethyl-nepenthone derivatives (4-series). **(B)** Binding mode for 17-nor-N-cyclopropylmethyl-nepenthone (compound **4**, colored in orange) at the agonist-binding site of KOR. The 7α-phenylcarbonyl group orientated very well inside the hydrophobic subpocket composed of residues Trp124^ECL2^, Leu135^3.29^, Val118^2.63^, Val134^3.28^, and Gln115^2.60^. Hydrogen bonding interactions are shown by yellow dotted lines.

Recently, Li et al. (2017) designed and synthesized two new series of nepenthone derivatives, among which compound **4** displayed the highest affinity (Ki = 0.4 ± 0.1 nM) and the highest selectivity (μ/κ = 339, δ/κ = 2,034) toward κOR (Figure 3A). Molecular docking operations were carried out to explore how compound **4** binds with the κOR (Figure 3B). The active κOR structure was built based on the crystal structure of the active murine μOR. Compound **4** interacts with conserved residues D^3.32^, I^6.51^, M^3.36^, Y^7.43^, W^6.48^, and Y^3.33^ at the active site of κOR. The carbonyl compound **4** formed a hydrogen bond with residue Q^2.60^, which induces the 7α-phenylcarbonyl group insert into the hydrophobic subpocket formed by residues W^ECL2^, L^3.29^, V^2.63^, V^3.28^, and Q^2.60^ of the receptor.

**Figure 4:**
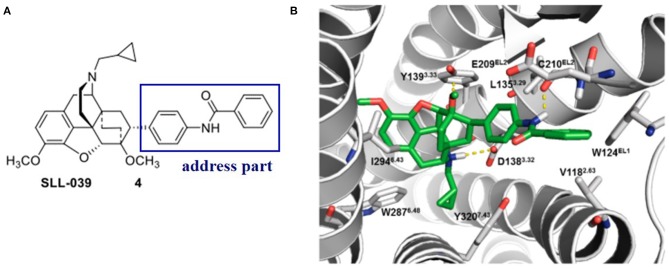
**(A)** Highly selective and potent κ opioid agonist SLL-039 (compound **4**) and its address part. **(B)** Putative binding mode of SLL-039 (in green) in the κ opioid receptor binding site. The benzamide phenyl group of SLL-039 was fit into a tight hydrophobic subpocket composed of Val118^2.63^, Trp124^EL1^, and Glu209^EL2^. Hydrogen bonding interactions are shown by yellow dotted lines.

Later on, Xiao et al. (2019) discovered a benzylamine derivative (compound **4**, **SLL-039**) as a highly potent and selective κ opioid agonist (κ, Ki = 0.47 nM, κ/μ = 682, κ/δ = 283) (Figure 4A). Also, molecular docking was used to explain the selectivity (Figure 4B). Molecular docking revealed three possible reasons for its high selectivity and activity. Firstly, the benzamide group could form a hydrogen bond with the Cys210^EL2^ of the receptor, which was not available in the μ opioid receptor. Secondly, the benzamide phenyl group of **SLL-039** fit into a tight hydrophobic subpocket composed of Val118^2.63^, Trp124^EL1^, and Glu209^EL2^ in the κOR receptor, which is regarded as a novel “address” subsite. Finally, the relative structural rigidity of benzamide carbonyl linker could produce conformational constraints to the structure and help it fit into the subpocket of κOR but is not able to fit the corresponding sites in other receptor subtypes.

In these studies, molecular docking operations offered a visual modeling of the interaction between ligands and receptors and thus played a key role in exploring the molecular mechanism of the subtype selectivity of these newly discovered compounds toward κOR.

#### The δ Opioid Receptor

**Figure 5:**
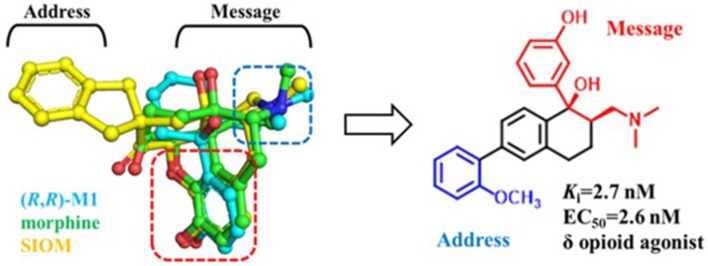
Design of (–)-6j using “message–address” concept. The nitrogen atom and phenol fragment of both (1R, 2R)-M1 (O-metabolite of Tramadol, colored in cyan) and SIOM (a δOR agonist, colored in yellow) overlapped very well after superimposing them together with morphine (colored in green). Different parts for these compounds are labeled according to the “message–address” concept.

**Figure 6:**
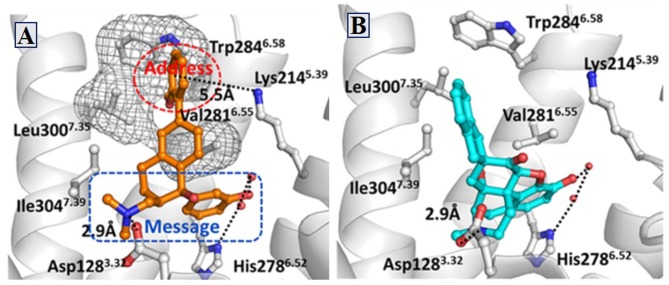
Binding structures of DOR-6j and DOR-SIOM obtained from molecular docking. **(A)** The binding mode of (–)-6j (colored in orange) at the agonist binding site of DOR. **(B)** The binding mode of SIOM (cyan color) at the DOR binding site. Important interactions are shown by black dotted lines.

Similarly, δOR also has an “address” subsite which is different from that of κOR. By revisiting the “message–address” concept, Shen et al. (2016) designed a serious of highly selective agonists of δOR from Tramadol. Among these new compounds, the compound (–)-6j displayed the best activity (EC_50_ = 2.6 nM) and δOR selectivity (more than 1,000-fold) (Shen et al., 2016) (Figure 5). Through molecular docking operations, it was revealed that the subsite of the “message” part in (–)-6j, i.e., the protonated nitrogen atom formed a strong salt bridge with residue D^3.32^ of δOR, while the phenolic group formed a strong hydrogen bond with the neighboring water molecule at the active site of the receptor. The “address” part of (–)-6j, the 6-ortho-methoxyphenyl, stretched into the hydrophobic pocket composed of residues L^7.35^, V^6.55^, and W^6.58^ of δOR and formed cation–π interaction with K^5.39^ (Figure 6). The binding mode of (–)-6j is consistent with the case of the δOR agonist SIOM (Portoghese et al., 1993). Overall, this study has proved that the “message–address” concept, together with the computational docking operations, can be applied successfully to design novel active compounds with better subtype selectivity.

### Molecular Dynamic Simulations to Study the Activation Mechanism

GPCRs form an important nexus between the extracellular stimuli and intracellular second messengers. Their conformations change frequently between ground state (R) and active state (R^*^) all the time. After binding with the agonist or antagonist, either the receptor's active state or its inactive state will be stabilized, and the ratio between different states will be altered. However, how an agonist induces the receptor to become active state is still not very clear. Although a number of GPCR structures are now available in the protein data bank (PDB), it is still not easy to illuminate the molecular nature of the conformational changes.

With the advancement of computing algorithms an powerful resources, computational studies on GPCRs are going deeper and more visible in the research community. As one of the powerful tools, the MD simulations have been used to figure out more puzzles about the GPCR and their interactions with downstream proteins at atomic level. By using MD techniques, researches can simulate the conformational evolvement of the receptor from a certain initial state on the basic of Newton mechanics and conduct the structural and functional studies. Up to now, many published studies have focused on the questions about the activation mechanism of opioid receptors (Kolinski and Filipek, 2008; Huang et al., 2015; Cheng et al., 2016; An et al., 2019). Among these studies, researchers are able to explore the biased activation mechanism toward a specific G protein (Schneider et al., 2016; Cheng et al., 2017).

#### The Activation Mechanism of Opioid Receptor

**Figure 7:**
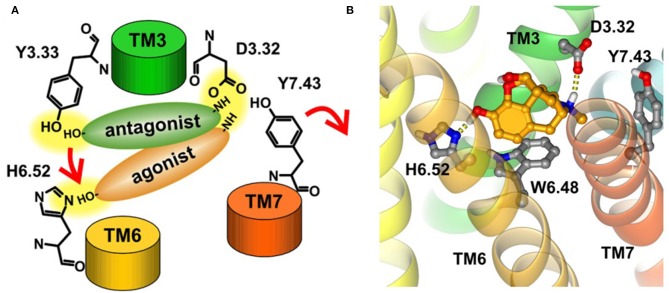
**(A)** Proposed binding modes of antagonists and agonists in opioid receptors. Red arrows indicate the suggested steps of agonist action. **(B)** Horizontal position of W^6.48^ interacting with morphine. The ligand is bound to H^6.52^ and D^3.32^.

Early in 2008, Kolinski and Filipek performed MD studies and analyses on the ligand–receptor complexes about two agonists (morphine and N-methyl-morphine), a selective antagonist β-funaltrexamine (β-FNA), and a non-selective antagonist naltrexone (NTX) through the trajectories of 20 ns MD simulations on the μ opioid receptor. In this work, they revealed the very first steps of receptor activation. For all the analyzed ligands, the protonated nitrogen atom was bound to D^3.32^ of the receptor, but their phenolic (C3) OH group interacted with a different binding anchor site. It seemed that antagonists tended to bind to Y^3.33^ residue, whereas agonists prefer H^6.52^ residue of the receptor. This indicated that the agonists could penetrate deeper into the active site of the receptor structure and induce a series of conformational transitions. During this movement, the hydrogen bond between D^3.32^ and Y^7.43^ inside the receptor was broken (Figure 7A), and these two residues turn to other neighbor partners, respectively. These findings suggested that the D^3.32^-Y^7.43^ interaction should be the important stabilizing factor for the inactive state of opioid receptors. In addition, the study also found that W^6.48^ acted as a rotamer toggle switch for the receptor activation. During the MD simulations, it was found that the agonists changed the rotamer of W^6.48^ to a horizontal position (perpendicular to TM6), while the antagonists maintained the initial vertical position of W^6.48^ (Figure 7B). However, the previously reported TM3–TM6 links inside the receptor were firmly maintained in the binding of both the agonists and antagonists. The researchers also indicated that the rotamer configurations of C^6.47^, W^6.48^, and F^6.52^ of the receptor were coupled with each other, modulating the bend angle of TM6 around the kink at P^6.50^. In 2015, Huang et al. (2015) indicated in their study the conformational movement of the transmembrane helix. At the cytoplasmic surface of the μOR, they observed large outward movement of TM6 (10 Å) and a slight inward shift of TM5 and TM7.

Meanwhile, Cheng et al. (2016) discovered that residues of E6.58 and I6.55 played a pivotal role in the activation of κOR. Fluctuation of I6.55 triggered the conformational change, immediately induced the motion of E6.58, and finally led to the rearrangement of each transmembrane helix. To better investigate the conformation dynamics, in 2019, An et al. (2019) carried out long-time Gaussian accelerated MD (GaMD) simulations on κOR binding with agonist MP1104 and antagonist JDTic. They found that the free of κOR was the most stable, while the active a κOR-apo would gradually change into inactive state. When κOR binds with agonists, some crucial motifs (DYYNM and CWxP) inside the receptor will be stabilized, increasing the ratio of active state in conformation equilibria. Antagonist binding with the receptor could not shake the inactive conformation, and these crucial motifs of the receptor were maintained well, although there was a stable intermediate (I) state between the active state and inactive state of the receptor (An et al., 2019).

#### The Mechanism of G Protein-Biased Activation

It is interesting to explore the conformational differences of the same receptor toward the binding of different G proteins. That is, the G protein-biased activation. To investigate the mechanism of G protein-biased activation, Cheng et al. constructed five μOR systems: the G protein-biased agonists TRV130 and BU72, antagonists β-FNA and NTX, and the free receptor (Cheng et al., 2017). According to their study, W^6.48^ and Y^7.43^ of μOR were proposed as a paired activation switch. These residues may play an important role in the binding of β-arrestin, thus regulating G protein signal transduction. As the first representative μOR ligand with G protein bias, TRV130 was found to directly interact with Y^7.43^ and make it closer to W^6.48^. Such interaction would stabilize the rotamer of W^6.48^ side chain, mostly at −70° and downregulate the binding of β-arrestin. Also, W^7.35^ was observed to be stabilized by a hydrophobic interaction. All these findings have been validated by experimental mutation studies (Hothersall et al., 2017; Sun et al., 2017).

Unlike traditional molecular docking, MD simulations can take good care of the conformational changes of the receptor. The only constraint of MD is the huge demand of computing resources. For a particular opioid receptor, including the G protein into the simulating system will definitely better simulate the whole process of ligand binding and receptor activation. However, it will also add about 50,000 atoms into the system, making the system very huge. Thus, such computing effort is very possibly undergoing in research labs, but still in the air of such publications. Also, it has been indicated that the timescale of activation for a GPCR receptor could be at the scale of microsecond or seconds (Dror et al., 2011), which will hopefully be revealed soon to the research community.

## Perspectives

Scientists have been making many research efforts to discover analgesics with less side effects, although the way looks not short. As experimental techniques become more matured, more and more crystal structures of opioid receptors and other GPCRs are quickly coming out. The availability of these new crystal structures in PDB at high resolutions is stimulating new rounds of virtual screening and activation mechanism study. Also, as the timescale of current MD simulations is increasing rapidly, some important conformational changes missed in the previous explorations may be captured and interpreted more reasonably for the opioid receptors in the near future. In addition, a number of new techniques, like enhanced sampling of MD simulations, have been developed and effectively applied to the studies on GPCRs. These techniques include targeted MD (Schlitter et al., 1994), steered MD (Isralewitz et al., 2001), and accelerated MD (Hamelberg et al., 2004; Miao et al., 2015). It is reasonable to expect that the molecular mechanisms of opioid receptors will be investigated and demonstrated more completely in the next couple of years.

## Author Contributions

BZ and WL wrote the manuscript. LS and WF revised the manuscript. All authors were involved in the preparation of the manuscript and approved the final version.

## Conflict of Interest

LS was employed by Shijiazhuang No. 4 Pharmaceutical Co., Ltd. The remaining authors declare that the research was conducted in the absence of any commercial or financial relationships that could be construed as a potential conflict of interest.
